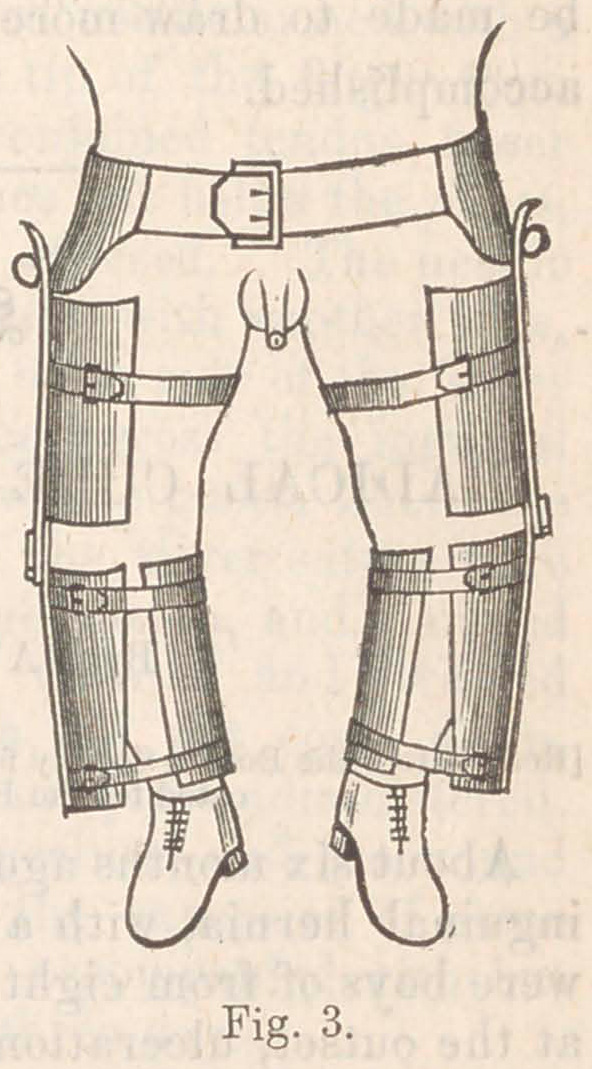# Improved Methods of Treatment in Deformities

**Published:** 1863-11

**Authors:** E. Andrews

**Affiliations:** Professor of Surgery in Chicago Medical College


ARTICLE XXXII.

### IMPROVED METHODS OF TREATMENT IN DEFORMITIES.

By E. ANDREWS, A.D., M.D., Professor of Surgery in Chicago Medical College.

In a former number, I gave an account of several improved methods of treatment in joint and spinal diseases. A number of important points were necessarily omitted in that essay for want of space. This article is designed to make good the deficiency. We will first consider the subject of

### ANCHYLOSIS OF THE KNEE.

This condition results from inflammation, in consequence of which plastic lymph is effused upon the articular surfaces, causing them to adhere firmly together and prevent all motion. In a few cases, the cartilages are removed by ulceration, and a bony union is formed. Authors commonly distinguish the two forms, as *false* and *true anchylosis.* The word anchylosis, however, as taken from the Greek, is equally applicable to both forms; and the use of the adjectives *false* and *true*, in such a connection, is awkward and ungraceful. *Anchylosis*, in Greek, simply refers to the bent or hooked form which stiffened limbs generally assume, and has no reference to the presence or absence of bony deposits. I prefer, therefore, to divide the affection into *fibrous* and *bony* anchylosis, rather than into *false* and *true.* Practically, the bony form is rarely found, ninety-nine cases out of a hundred being of the fibrous variety. During the inflammatory stage, the patient seeks a partial relief of his pain by placing the limb in a flexed position; hence, we usually find the knee not only stiff, but flexed at an angle, or even an acute angle.

There are two objects, therefore, to be gained by the treatment: first, to straighten the limb so that the foot may be brought to the ground; and, secondly, to restore the mobility of the joint. The resistance to the straightening process, in fibrous anchylosis, is maintained by three tissues, viz.: the flexor muscles and tendons, the shortened ligaments, and the new fibrous tissue connecting the inner faces of the joint to each other. All these obstacles will yield to steady tension; and, if the patient and surgeon think best, the limb may be perfectly straightened without any operative procedure.

In all the more difficult cases, however, the flexor tendons oppose so much resistance that it is, practically, much the best to divide them at the outset with a tenotome. We have then simply to overcome the resistance of the shortened ligaments and of the new tissue in the anterior of the joint. For this purpose, extend the leg until the tendons of the hamstrings are quite tense, and then divide them, taking care to avoid the peroneal nerve by the tendon of the biceps. The patient should be well under the influence of an anæsthetic; and, after the tendons are severed, moderate efforts may be made to straighten the limb by force. Great violence, however, should not be used, as serious and even fatal results have followed such a course. If the adhesions refuse to yield to a moderate force suddenly applied, we must next resort to gradual extension.

Fig. 1.

For this purpose, I use the instrument shown in Fig. 1. This consists of a half-armor, covering the posterior portion of the limb and extending from the ankle to the middle of the nates. An extension-brace, formed by a tube and a screw, passes across the angle, for the purpose of applying a powerful force in obstinate cases; but, in more tractable limbs, the brace is removed, and the force obtained by means of the strong rubber springs on either side of the knee, attached to the upper edges of the apparatus. The knee must be kept in by a cloth knee-cap firmly strapped down, and the leg and thigh bound in by a band or two of cloth or of adhesive-straps. The principle of this splint has been in use a long time, but one or two points are new. The prolongation of the thigh-piece upward upon the nates is to get a firm bearing upon the ischium so as to avoid pressing the upper end upon the sciatic nerve, which is apt to occur in the old form figured in the text-books. Care must be taken that the edge of the instrument on the outer border of the knee does not press upon the peroneal nerve and paralyze the dorsum of the foot. If the brace is used for extension, the nut must be tightened a little three or four times a day, until the joint is quite straight. In milder cases, the brace may be removed and rubber springs left to work alone, simply being tightened a little once in three or four days. The best material for construction is sheet-brass; but, if preferred, ordinary sheet-tin will answer, and a common tinner can construct every part of it, except the nut, screw, and rubber springs.

The straightening may be accomplished commonly within two months, meanwhile daily motions of extension and flexion should be made as diligently as the sensitiveness of the joint will permit, in order to restore the mobility. This restoration of the power of motion is a slower and more tedious process than the mere straightening; but, nevertheless, if the passive exercise is continued long enough, the result is tolerably certain in favorable cases. If the anchylosis is bony, the straightening is still possible in the same way as before; but, practically, the difficulties are so great that operative interference is preferable. A wedge-shaped piece of bone may be removed in such instances, and the leg then brought down.

If the anchylosis has occurred during early childhood, with so much flexion that the foot cannot be used, atrophy takes place, and the hope of restoring a useful limb, in adult life, is sometimes futile. If the leg is a serious encumbrance, in such cases amputation must be performed.

TREATMENT OF TALIPES WITHOUT TENOTOMY.

In my former article, I referred to the fact, that nearly all cases of talipes may be cured without cutting the tendons, and that some surgeons have ceased to perform that operation in ordinary cases. As the dressings required for this mode of treatment are more easily understood by the help of an engraving, I have had a cut prepared for the sake of illustrating my meaning, and may be excused for repeating the substance of the former explanation of their application. The fundamental maxim in these cases is this: *Every distorted joint may be made to return to its normal position by steady and long continued traction.* The principle of the management of talipes without tenotomy is, therefore, very simple; but the successful application of it depends upon the patience, faithfulness, and ingenuity of the surgeon. There are also a few instances where the practical difficulties render the principle inapplicable. The appliances must be prepared by the surgeon for each particular patient, and varied to suit the peculiarities of the case;

Fig. 2.

and the materials for them consist mainly of adhesive-plaster and elastic webbing. The following description may serve to convey the general idea. We will suppose it to be a case of talipes varus. The first thing to be done is to secure two firm points of traction, which will not hurt the patient. For the first, we envelope the foot in bands of adhesive-plaster, carefully adjusted, bringing their free ends under the sole and up the outer side. They are there gathered in one, two, or three groups, or sometimes all attached to a small rod running parallel to the outer border of the foot. The second point of tension is easily made by attaching broad adhesive-straps to the upper part of the outer side of the leg. It is convenient to arm the lower extremities of these with light buckles. The upper and lower adhesive-straps are now connected by from one to three strips of elastic webbing, which, of course, pass over the outer maleolus and tend to draw the foot to its position. A small cushion should be placed upon the maleolar region to receive the pressure of the bands. Thus prepared, let the elastics be buckled to a very gentle tension for the first few days, until the skin becomes accustomed to the presence of the apparatus, after which, they may be gradually tightened. The tension being moderately kept up day and night occasions very little pain, and the contracted parts slowly yield until the foot assumes a perfect position. Many weeks are often consumed in the treatment; but if the parents are intelligent, the surgeon need not see the child very often after the first twelve days.

Many other applications of these principles will readily suggest themselves to the ingenious practitioner, but which cannot be detailed in this brief essay.

We may truly say that, for those afflicted with spinal curvature, hip-disease, inflammation of the knee, or club-foot, a new era has dawned; and vast numbers of cases, supposed by our predecessors to be hopeless, will, in our day, be restored to soundness and perfect form.

The engraver has, in the above cut, misrepresented the malleolar cushion, causing it to look like a roller of solid wood. Of course, no one will be misled by the error. A good club-foot shoe can be made to accomplish the cure, but not so easily as the elastic bands.

### BOW-LEGS.

One of the most difficult and vexatious deformities ever brought up for treatment is bow-legs. It is caused by rickets, in some instances, and in others by too early efforts of the child to walk, by which the tibia is flexed with the convexity outward, and the whole limb assumes a bow form. The principal curvature is usually at the point where the upper part of the tibia joins the epiphysis. There is often a slight degree of this deformity in young children, which disappears without treatment before the child reaches the fourth year of its age; but in more aggravated cases, it continues and constitutes a permanent blemish. After a variety of troublesome experiments, I have devised the apparatus represented in Fig. 3, which answers the purpose perfectly. A spring-steel band passes partly around the waist being left open in front where the vacancy is filled by straps and a buckle. On each side a projection of the steel extends downward until it overlaps the trochanter major. To each projection a steel strap is articulated, extending downward to the knee, and carrying an armor which embraces the outer half of the thigh. At the knee another strap articulates with a similar half-armor for the outer side of the leg. A narrow piece of armor is also made to fit the inner side of the leg. The joints of the instrument must be made to come accurately opposite the hip and knee joints; and those opposite the knee, while they move easily backward and forward, must firmly resist any lateral flexion. For this purpose, the rivet must have a broad strong head. The whole must be nicely covered and padded. If now the band is buckled around the waist and another be passed around the middle of each thigh, it will be found that while the limb applies very well, as far down as the knee, it there leaves the armor and curves inward. The proper pieces must now be placed along the inner side of the leg, and, by means of straps and buckles, be drawn outward towards the outside pieces. The spring of the steel keeps up a constant elastic tension; and, by daily tightening the straps, the limb will be slowly brought back to a perfect form.

Fig. 3.

Care must be taken not to apply the straps too tightly at first, otherwise the skin will abrade and ulcerate, and the whole treatment be delayed. It will be sufficient if, during the first ten days, the instrument be worn very lightly,—just pressing enough to accustom the skin to its presence, after which, it can be made to draw more powerfully, until the object in view is accomplished.

## Figures and Tables

**Fig. 1. f1:**
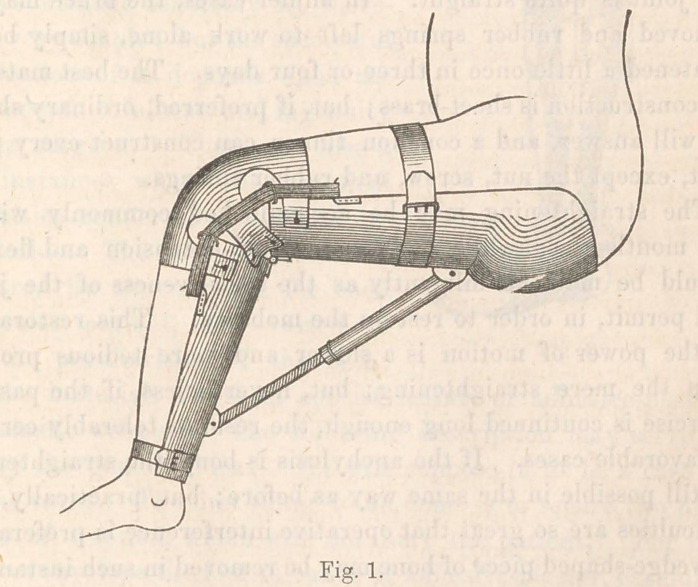


**Fig. 2. f2:**
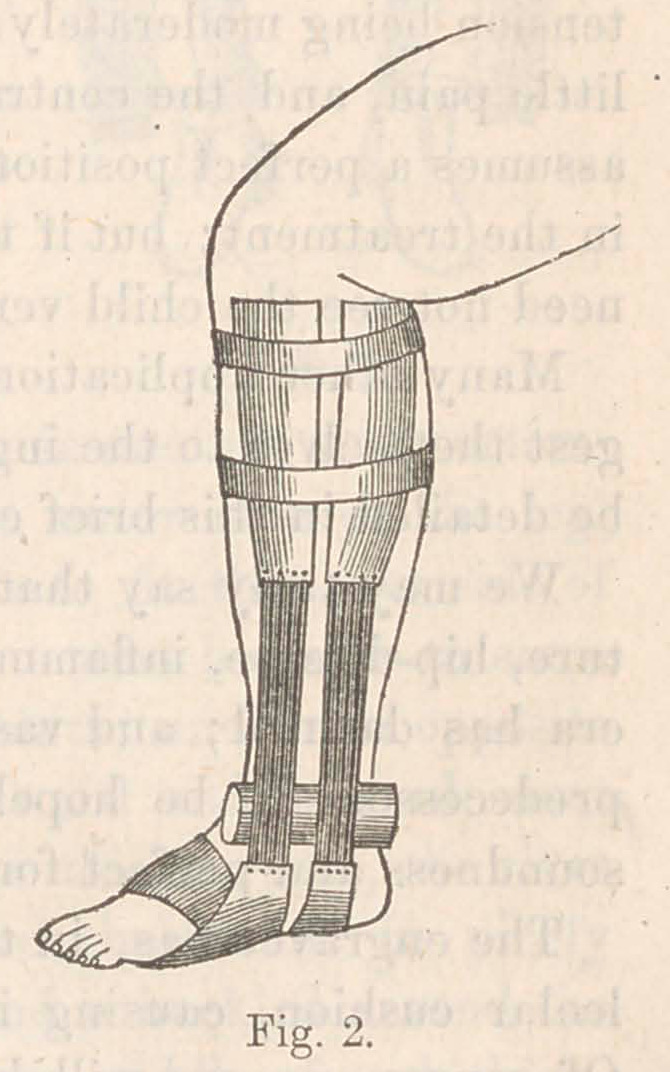


**Fig. 3. f3:**